# Faster Recovery of Internal Ophthalmoplegia than External Ophthalmoplegia in a Miller Fisher Variant of Guillain-Barre Syndrome

**DOI:** 10.1155/2020/7258327

**Published:** 2020-04-12

**Authors:** Golla Abhinav, Jorge Gamez, Michael C. Yang, Tetyana Vaysman, Michelle von Gunten, Antonio Liu

**Affiliations:** ^1^Department of Neurology, Adventist Health White Memorial, Los Angeles, California, USA; ^2^Department of Internal Medicine, University of Maryland, Cheverly, Maryland, USA

## Abstract

We present a case of classic Miller Fisher Syndrome (MFS) variant of Guillain-Barre Syndrome (GBS) with detailed description in the difference between the internal and external ophthalmoplegia. They are different in their onset, duration, and recovery.

## 1. Introduction

MFS variant of GBS is associated with both the internal and external ophthalmoplegia [1]. Prior studies have noted that the defect in pupillary reactivity can vary with time [2]. With the pupillometer (NeurOptics NPi**®**-200), it is now possible to accurately and consistently measure pupillary size and reactivity. Ultimately, this device allows clinicians to objectively quantify pupillary function with the standardized Neurological Pupil Index (NPi) Pupil Reactivity Assessment Scale.

The pupillometer is an easy-to-use, handheld device that can be used at bedside. It is loaded with a SmartGuard cartridge and held up to the patient's eye. The device accurately and consistently measures pupillary size and reactivity speed. The Neurological Pupil Index (NPi) Pupil Reactivity Assessment Scale scores range from 0 to 4.9. A score of 0 represents a nonreactive, immeasurable, or atypical response. A score of less than 3.0 indicates abnormal (“sluggish”) reactivity. A score of 3.0-4.9 indicates normal (“brisk”) reactivity. Additionally, a NPi score difference that is greater than or equal to 0.7 between the right- and left-eye measurements suggests a pupillary abnormality.

Currently, the literature describing the rate of recovery of the internal and external ophthalmoplegia in MFS is scarce. We present a case of antibody-proven MFS with the classic pattern of descending weakness. Notably, our patient's internal ophthalmoplegia developed prior to external ophthalmoplegia and also resolved much earlier.

## 2. Case Presentation

A 44-year-old right-hand-dominant male with no significant past medical history presented to the emergency department with two days of difficulty speaking and loss of balance, stating “my voice is just different.” The patient endorsed having severe diarrhea two weeks prior and had also received the influenza vaccine six months prior to admission. Upon presentation, the patient's vital signs were within normal limits. Upon interview, examiners did not appreciate any aphasia, but the patient's speech possessed a notable nasal quality. Upon physical examination, the patient required major assistance to ambulate. All physical symptoms were acute in nature; he was previously working, ambulating, and completing activities of daily living without any issues.

**Figure 1:**
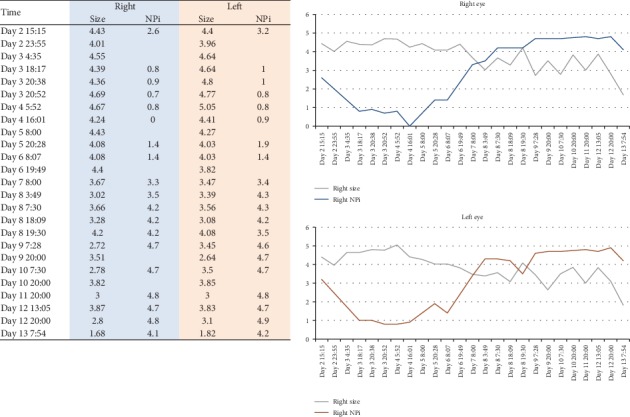
Daily pupillometry measurements. Relationship between pupil size and NPi.

Initial exam of extraocular movements revealed minimal deficits in left-eye abduction and horizontal nystagmus that changed direction with lateral gaze in either direction. Over the next three days, the patient's minor ocular movement deficits progressed into severe external ophthalmoplegia in all directions. During this time, he also developed bilateral ptosis. Pupillary size and reactivity were measured daily using the pupillometer. During hospital day one and two, pupillary function remained relatively normal (right NPi 2.6 “borderline sluggish,” left NPi 3.2 “brisk”). However, on hospital day three, pupillometer readings suggested that the patient's pupils were “sluggish” bilaterally (right NPi 0.7, left NPi 0.8) (Figure 1).

The table shows the recorded pupil size and corresponding NPi score over the course of the patient's admission. The adjacent graphs represent the recorded measurements through time (the gray lines indicate pupil size (in mm), blue line indicates right NPi, and red line indicates left NPi).

Throughout the hospital course, the patient's nasal tone remained unchanged, but he developed moderate to severe dysarthria and minimal to moderate dysphagia. The patient's extremity and truncal ataxia continued to worsen, and he subsequently required moderate assistance with a walker to ambulate. Sensation to light touch, temperature (ice examination), and proprioception remained intact. However, he reported tingling and “skin tightness” that persisted for over one week. The patient never developed urinary or bowel incontinence. He denied shortness of breath, maintained a normal vital capacity, and exhibited normal arterial blood gas studies.

Ganglioside antibody panel was sent out on hospital day one and resulted on hospital day ten, which was remarkable for elevated antibody levels (Asialo-GM1 Ab 279, GD1a Ab 52, and GQ1b Ab 273). Due to a high suspicion for an autoimmune neuromuscular disease, plasma exchange was initiated on hospital day three. A total of five plasmapheresis treatments were administered. The patient developed orthostatic hypotension on several occasions that led to two syncopal episodes, both within an hour of plasma exchange treatment. Pupillary reactivity recovered within four days of symptom onset (by hospital day seven); however, it took several weeks for external ophthalmoplegia to resolve (Figure 1). The patient was discharged home on hospital day twenty. At the time of discharge, his external ophthalmoplegia persisted with only partial recovery. Six weeks after discharge, the patient's symptoms had completely resolved and antibody levels had normalized (Asialo-GM1 Ab 50, GD1a Ab 18, and GQ1b Ab 48).

## 3. Discussion

Miller Fisher Syndrome (MFS) is a rare variant of Guillain-Barre Syndrome (GBS), occurring in 1-7% of GBS cases worldwide [3] and 5% of GBS cases in Western countries [4]. Early ocular findings of MFS and GBS include ophthalmoplegia, diplopia, and pupillary abnormalities (internal ophthalmoplegia)—all of which have been well described in the literature. However, at the time of authorship, the natural history of pupillary deficits and external ophthalmoplegia in MFS has not yet been objectively described in the literature.

The literature contains many references of MFS presentation, with both a typical triad of ophthalmoplegia, areflexia, and ataxia and atypical variants of MFS different in combination of symptoms. Kaymakamzade et al. have reported an interesting case of atypical MFS in a 17-year-old male patient, confirmed by raised titers of anti-GQ1b antibodies with an early onset of external ophthalmoplegia following by internal ophthalmoplegia and characterized by mydriasis and decreased reactivity to light [5]. Lopez et al. [6] have described a case of a 74-year-old woman with a sole presentation of internal ophthalmoplegia evidenced by nonreactive midsized pupils with preserved visual acuity as initial manifestation of MFS. The patient developed external ophthalmoplegia, mild ataxia, and hyporeflexia by the second day of the initial presentation and full resolution in two months [6]. These cases are interesting in demonstrating sequence of presentation, whereas out investigation is unique in focusing on difference in onset, duration, and recovery rate between the internal and external ophthalmoplegia.

This case report demonstrates that the internal and external ophthalmoplegia can occur together in MFS but are independent of each other in terms of onset, severity, and duration. Man [1] describes the case of a 46-year-old patient with total internal and external ophthalmoplegia as the initial presenting symptoms of MFS, confirmed with serum-positive anti-GQ1b antibodies. Serum anti-GQ1b antibodies are associated with GBS and MFS. GQ1b is a ganglioside that is commonly found in cell membranes of cranial nerves that innervate extraocular muscles (oculomotor, trochlear, and abducens nerve), confirmed by immunohistochemical studies [7]. Evidence strongly suggests that the ophthalmoparesis in MFS results from a direct action of anti-GQ1b antibodies on the presynaptic neuromuscular junction (NMJ) between cranial nerves and extraocular muscles [8]. Anti-GQ1b antibodies bind to presynaptic receptors which trigger a large release of acetylcholine and ultimately impair NMJ function [8]. Additionally, GQ1b gangliosides are also found in the ciliary ganglion, which is a presynaptic ganglion responsible for pupillary sphincter and ciliary muscle control [8]. These mechanisms may explain the prevalence of the internal and external ophthalmoplegia in MFS and GBS.

The use of a pupillometer in our presented case allowed for objective measurements of pupillary size and reactivity over the disease course of MFS. The patient's pupillary reactivity worsened acutely on hospital day three and resolved after four days (hospital day seven) (Figure 1). External ophthalmoplegia reached its nadir on hospital day seven, continued to persist on hospital day twenty with only minor improvement, and eventually resolved after several weeks. The patient's symptoms were completely resolved by the six-week follow-up appointment after discharge. This case report of Miller Fisher Syndrome (MFS), with positive anti-GQ1b antibodies, objectively documents the natural history of the internal and external ophthalmoplegia and demonstrates that these two entities can be independent of each other with regard to disease onset, severity, and duration.
